# Bioinspired Drilling for Extraterrestrial Applications

**DOI:** 10.3390/biomimetics10110752

**Published:** 2025-11-07

**Authors:** Gal-Erdene Battsengel, Noune Melkoumian, David Harvey, Rini Akmeliawati

**Affiliations:** 1 Discipline of Mining and Petroleum Engineering, School of Chemical Engineering, The University of Adelaide, Adelaide 5005, Australia; noune.melkoumian@adelaide.edu.au; 2 School of Electrical and Mechanical Engineering, The University of Adelaide, Adelaide 5005, Australia; david.harvey@adelaide.edu.au (D.H.);

**Keywords:** bioinspired drilling, extraterrestrial drilling, extraterrestrial exploration, mining

## Abstract

This review presents the novel synthesis of nature-inspired drilling strategies specifically tailored for extraterrestrial environments, where conventional technologies fail under the environmental conditions and power and mass constraints. Biomimetic drilling, inspired by insects, mollusks, reptiles, and other organisms, offers novel solutions for extraterrestrial subsurface exploration. Numerous organisms efficiently penetrate materials with low energy, using little force, and adapt to flexible substrates, which are essential capabilities for use off this planet. Traditional rotary and percussive drills do not function well under microgravity, at the end of the temperature spectrum, or in low energy and mass environments, such as landers which are typically under 300 kg and 200 W of power available. Nature-inspired approaches such as the reciprocating carpenter bee style have been shown to reduce overhead forces by as much as 50%; clam-like fluidization reduces drag by 90%; and sandfish-inspired methods improve mobility in granular media by 40%. These also improve the in situ resource utilization (ISRU) approaches for efficient sampling, water ice extraction, and planetary surface operations. This paper focuses on bio-drilling with other biological models, their engineering analogs, and exploration models for off-Earth use. Based on this synthesis, the paper recommends prioritizing dual-reciprocating and oscillatory mechanisms for near-term missions, while pursuing hybrid, AI-driven, and wear-resistant designs for long-term exploration. These approaches will help to improve penetration efficiency, reduce power demands, and extend the drilling system’s lifespan in challenging extraterrestrial environments.

## 1. Introduction

The exploration of extraterrestrial bodies, ranging from the moon and Mars to asteroids and beyond, necessitates powerful drilling technologies capable of penetrating unfamiliar and frequently extreme subsurface conditions. Conventional drilling technologies are widely utilized on Earth in industries such as oil and gas extraction, mining, and geotechnical engineering [1]. However, direct application of these technologies to space missions presents numerous hurdles, associated with reduced gravity, extreme temperature changes, limited accessible power, abrasive regolith, and strict mass limitations [2,3]. For instance, payload delivery costs to low Earth orbit (LEO) are typically around $2700 to $5000 per kilogram using current launch systems (e.g., SpaceX Falcon 9) and can exceed USD $20,000 per kilogram for Mars missions [4,5]. These limitations make it essential to minimize the mass of equipment, including drilling systems, without compromising their functionality. Beyond sampling and site characterization, such drilling systems also play a crucial role in supporting in situ resource utilization (ISRU), which aims to extract and process local materials such as water ice, regolith, and volatiles for life support, fuel production, and construction for long-duration missions [6].

Bioinspired and biomimetic designs have gained popularity in the engineering community in recent years due to their potential to overcome complicated technological problems [7]. Biomimicry is about understanding biological structures, systems, and processes and then using their fundamental principles to create new technologies. Nature has an extensive portfolio of optimized, efficient systems that have evolved over millions of years. These natural systems often provide lightweight yet robust designs, energy efficiency, and distinct mechanical qualities that can be directly applied to engineering solutions. Examples of bioinspiration in many fields of engineering are presented, such as gecko-inspired adhesives and bird-inspired aerodynamics for unmanned aerial vehicles [8,9]. In the field of drilling, organisms such as wood wasps, beetles, and other boring insects have unique penetration methods that can show how to optimize drilling in tough conditions, reduce tool wear, and save energy [10].

Conventional drilling methods use rotary or percussive devices, such as a weighted drill string, high-torque motor, or hammering action to break up and remove rock or soil [1,11]. While these strategies have proven to be successful on Earth, they present significant logistical challenges in space. Previous publications [2,3] have argued that that it is difficult to induce significant downforce in a low-gravity environment, and the high-power requirements of a rotating system may surpass a space mission’s limited energy budget. Percussion drilling causes detrimental vibrations and necessitates significant mechanical support, adding mass to the spacecraft. Furthermore, regulating cutting and preventing clogs in regolith or icy conditions pose significant engineering challenges.

To address these restrictions, biomimetic drilling systems look to nature for solutions [11]. For example, certain insect ovipositors use the reciprocating action of micro teeth to drill through wood or other substrates, significantly lowering the required axial load while still penetrating the material [12]. Similarly, some marine species’ peculiar helical shapes provide an effective drilling process that reduces friction losses while maintaining a steady tool path. Engineers aim to reduce power consumption, weight, and vibration and achieve better control over debris management by researching and reproducing biological strategies that could apply to extraterrestrial environments [13].

**Figure 1:**
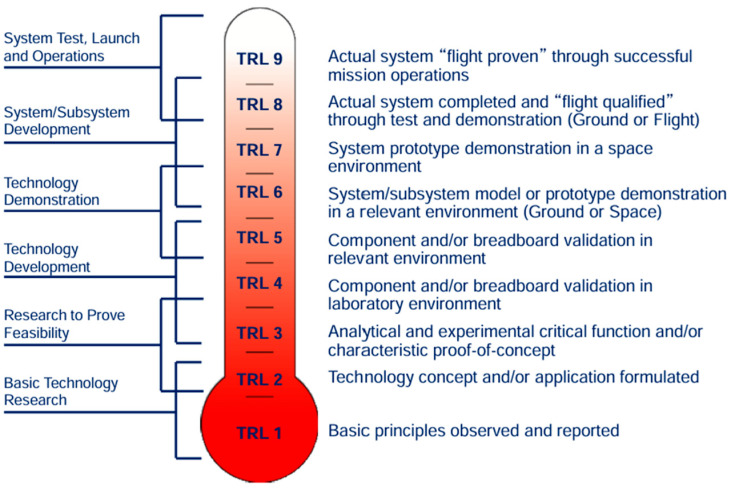
Maturity levels based on Technology Readiness Level [14].

This paper presents a critical review on biomimetic drilling principles and the possibility of applying them in space. It starts with a discussion of the biological systems that motivate novel drilling techniques, including examples from the wood wasp, locust, earthworm, and razor clam. The models are studied in space environments, such as vacuum, radiation, and low gravity. Regarding the Technology Readiness Level (TRL), which is a NASA-developed scale from 1 to 9 used to assess the maturity of a technology, each level description can be found in Figure 1 [14]. The paper then contrasts the biological approaches with key requirements for planetary subsurface access and ends by suggesting some directions for high-altitude drilling systems of the next generation, focusing on energy consumption, mechanical field-adaptability, and durability in hostile environments.

## 2. Bioinspiration in Engineering

**Figure 2:**
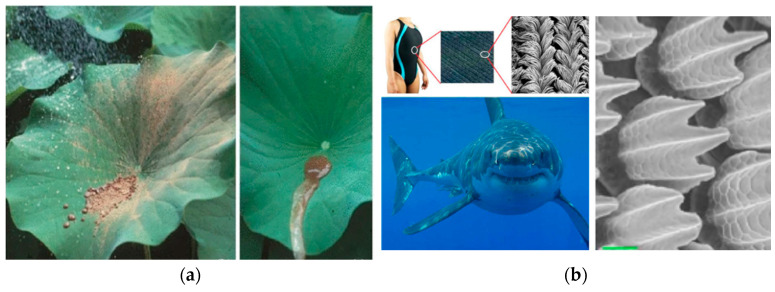
(**a**) Dirt particles are washed off with pure water from lotus plant [17]. (**b**) Dermal denticles inspired by shark skin [18,19,20].

Bioinspired engineering applies principles from nature to solve technical challenges, translating biological strategies into materials, devices, and processes across scales [7]. These solutions often provide high efficiency, adaptability, and resilience—qualities essential in space exploration. Figure 2 illustrates two widely known examples: Figure 2a the lotus leaf, which inspired self-cleaning surfaces; Figure 2b shark skin denticles, which informed drag-reducing designs for swimsuits and aircraft [15,16].

**Figure 3:**
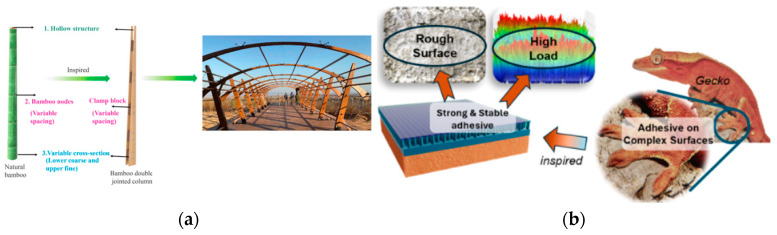
(**a**) Bamboo-inspired design in structural engineering [21]. (**b**) Representation of gecko-inspired adhesive [22].

Likewise, Figure 3a shows bamboo-inspired structures and Figure 3b gecko-inspired adhesives, which combine lightweight strength with strong yet reversible attachment. Such principles are directly relevant to extraterrestrial drilling, where low mass, minimal power, and durability are mission-critical. The following sections examine specific biological models, namely wood wasp ovipositors, clam fluidization, and sandfish undulation, that have inspired novel drilling systems for planetary environments.

## 3. Review of Bioinspired Drills

Throughout this review, the terms “biomimicry” and “biomimetics” are used; these may occasionally be used interchangeably but differ subtly in context. Biomimicry in design frequently focuses on imitating nature’s methods and systems for sustainability (in the study of architecture or ecology) using a model that provides an influence, in a similar way to samples found in processes. On the other hand, biomimetics describes the practice of replicating a mechanism or function found in biology to solve various problems, rather than describing a study of living systems as such. Due to the applied nature of extraterrestrial drilling, most of these are in the domain of biomimetics.

Many animals in nature use granular materials like sand and soils to find food, dig burrows, and hide to avoid predators [23]. The tactics of biological burrowing processes are classified into two orientations for cutting the surface, i.e., vertical and horizontal, and each biomimetic methodology is thoroughly discussed under this section.

### 3.1. Vertical Penetration

#### 3.1.1. Wood Wasp-Inspired Models

**Figure 4:**
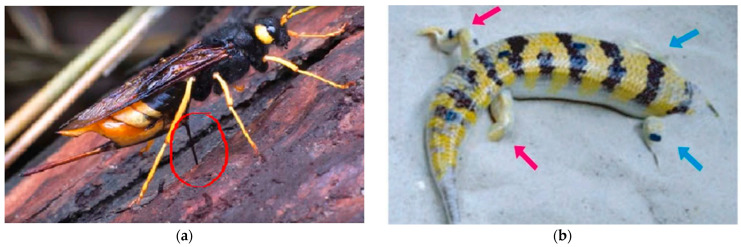
(**a**) Wood wasp depositing its eggs into a tree [25]. (**b**) Sandfish diving into sand [26].

Research into wood wasp-inspired drilling processes has resulted in important breakthroughs for extraterrestrial subsurface exploration. A study presented an innovative concept for the wood wasp ovipositor (Figure 4a), which uses a double-valve reciprocating motion to drill effectively without requiring a large external downward force [12,24]. This technique has been applied to a biomimetic robotic drill, which has demonstrated a higher energy efficiency and penetrating capabilities than traditional rotary and percussive drills [25].

The wood wasp-inspired drill could achieve a drilling speed of about 1 mm/min in the early stages and up to 1.5 mm/min as drilling progressed [12]. This performance is attributed to the staggered arrangement of the push teeth, which equally distribute cutting pressures while conveying chips out of the drilled hole via designated grooves. Unlike typical drilling methods that rely on applied force, the wood wasp drilling mechanism creates traction internally between the reciprocating valves, avoiding the requirement for large axial loads [27].

**Figure 5:**
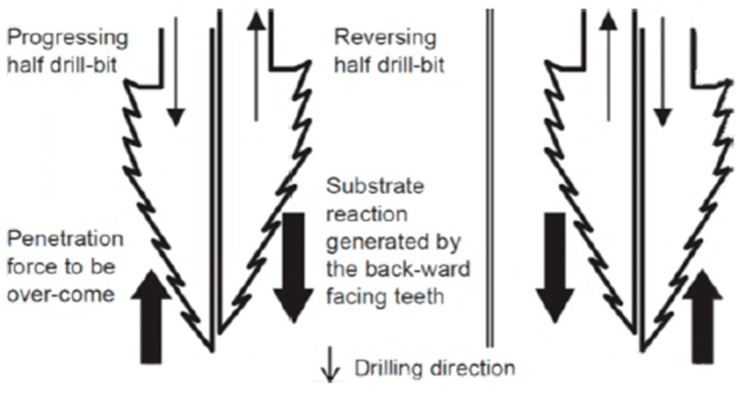
Initial diagram of DRD mechanism [28].

Laboratory studies have demonstrated that the double-reciprocating drilling (DRD) mechanism inspired by the wood wasp may function with 50% less top force than rotary drilling approaches, making it ideal for low-gravity extraterrestrial conditions [12,24]. Below, Figure 5 illustrates the initial concept of the drill. Furthermore, energy consumption was greatly reduced due to this design, with some prototypes requiring only 3 W of power to achieve a penetration depth of up to 2 m [12]. This efficiency is especially beneficial for space missions, where power supply constraints are a major problem.

The drill bit design was further refined based on the morphology of the wood wasp model to optimize the cutting profile and increase the penetration effectiveness. The study [29] found that biomimetic drill bits with spiral teeth and diamond cross-sections improve cutting stability and debris removal capabilities, lowering the risk of tool entrapment in the granular weathering layer of the simulants. Comparative experiments have revealed that this design can increase the penetration rate by 30% over traditional straight-cut drill bits while retaining structural integrity under different substrate conditions.

The versatility of the wood wasp-inspired drilling technology extends beyond extraterrestrial sampling and into biomedical applications. Studies on the ovipositor’s flexibility have shown that it can be bent to a radius of 0.5 mm without breaking, making it a perfect model for minimally invasive medical probes. According to a recent work, this flexibility enables trajectory modifications during underground drilling, minimizing the possibility of impediments and enhancing navigation in a heterogeneous terrain [13,30].

**Figure 6:**
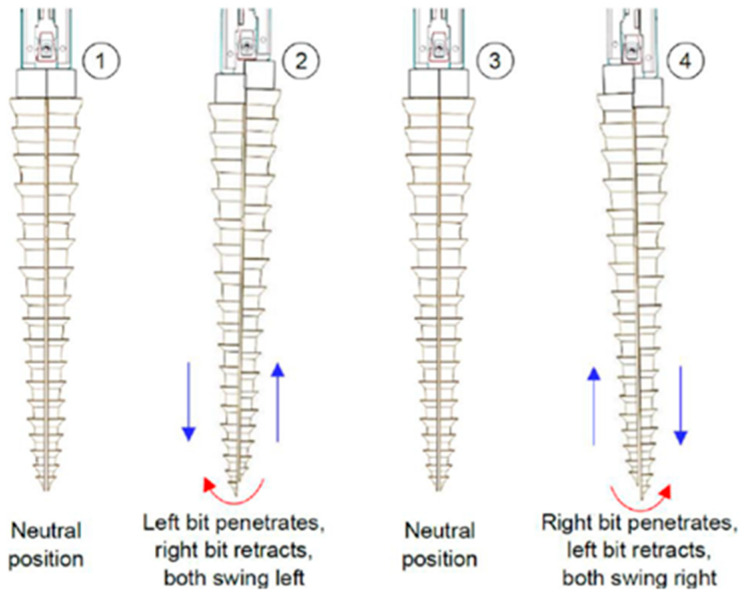
Drilling sequence showing each step and its motion [27].

Recent improvements in drilling mechanisms inspired by the wood wasp use hybrid reciprocating–oscillating technology to increase penetration efficiency, as presented in Figure 6. The double-reciprocating–oscillating drill (DROD) with penetration rates exceeding 2.5 mm/min in JSC-Mars-1 regolith and optimized drive control achieves up to 25% greater penetration depth than earlier designs under the equivalent energy input and substrate conditions [25]. Furthermore, studies on drilling through a Martian regolith simulant demonstrated that the DROD system reduced the drilling time by around 40% while using a minimal amount of energy. The publication underlined that advancements in drill bit morphology and drive control are projected to increase efficiency, ensuring that these biomimetic systems work reliably in extreme settings such as the moon, Mars, and asteroids [13,30]. To date, wood wasp-inspired dual-reciprocating mechanisms have not yet been used in space missions. However, they have undergone multiple terrestrial trials using Martian and lunar regolith simulants (e.g., JSC-Mars-1 and LHS-1) from 2019 to 2023. Tests demonstrated their stable performance at low axial loads and promising energy efficiency under simulated Martian gravity, but challenges such as actuator complexity and bit wear remain to be addressed [25,27].

One of the most remarkable advancements in bioinspired extraterrestrial drilling is the construction of the Integrated Acquisition Mechanism (IAM), which represents a hybrid evolution of the DRD system. Unlike conventional drilling systems which recover individual cores, IAM uses a rotating sleeve containing sample chambers to acquire and store multiple layers in sequence [31]. This allows for an extremely efficient science return, especially on time-limited, power-constrained, and mobility-limited missions. Laboratory validation of IAM in Martian regolith simulants showed that the retrieval rate could be increased by a factor of almost two, with an energy consumption below 10 W for the compacted substrates as well. The system also demonstrated an enhanced resistance to bit clogging and tool jamming, which are typical issues in dry abrasive conditions. IAM allows for a multi-depth stratified sampling and it has been designed to manage the requirement of finer-grained geotechnical and geochemical studies in planetary exploration [31]. The modularity of IAM also enables it to be used on robotic arms or lander-mounted platforms, making it even more versatile for future Mars and lunar missions. Based on all of the above-mentioned studies it can be evaluated that this concept is at a TRL of 4–5, as the components have been validated in laboratory environments with limited system integration.

#### 3.1.2. Locust-Inspired Models

**Figure 7:**
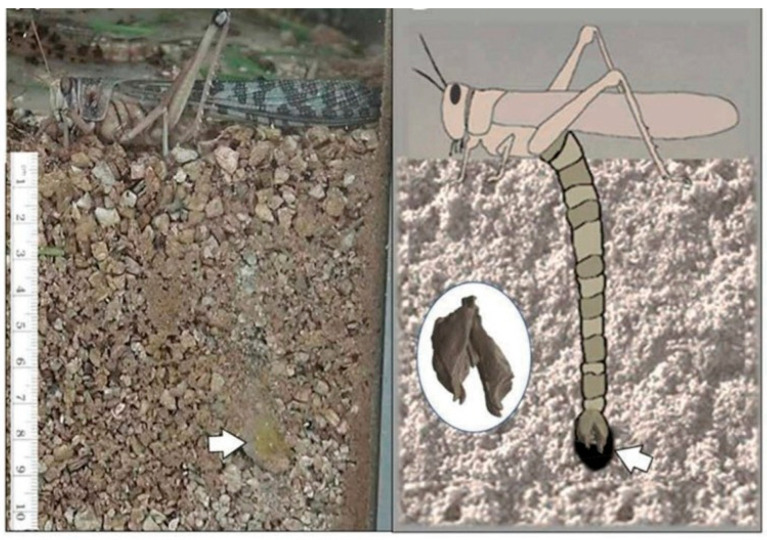
The process of laying eggs by a female locust [34].

Research on locust-inspired drilling methods sheds light on the biomechanics of efficient and energy-saving penetration devices. According to [32], the locust ovipositor is a specialized organ used by female locusts to deposit eggs, as illustrated in Figure 7, and can be utilized as a model for a biomimetic drill due to its capacity to pierce hard materials with low force. The female locust’s ovipositor is made up of two reciprocating valves that move in an asymmetric manner, allowing for a regulated excavation with a minimal resistance [33]. This method has been used in novel robotic systems intended for planetary drilling, where low energy consumption and adaptability to changing terrain are essential.

**Figure 8:**
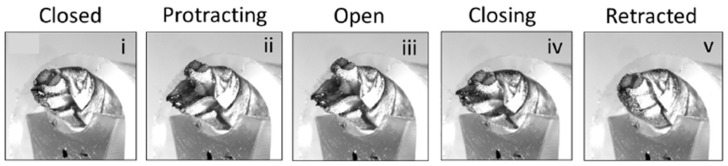
Close view of the valves with their opening and closing cycle [37].

Experimental investigations on the mechanical performance of the ovipositor revealed that the dorsal and ventral valves provide distinct functions during penetration. The dorsal valve offers stability and anchoring, whereas the ventral valve carries out the major cutting and gradually reaches deeper into the stroma [35]. The close-up view image in Figure 8 illustrates the opening and closing cycles. High-resolution imaging and material testing revealed that the valves are strengthened with a unique cuticle composition that increases their wear resistance and durability [36]. Incorporating this approach into a robotic drilling system resulted in the creation of an autonomous penetration device that employs a reciprocating motion to reduce the frictional resistance and improve the efficiency of drilling in granular and compacted soils [37].

Laboratory tests were conducted using a biomimetic drilling prototype based on the locust ovipositor, which dramatically reduced the necessary axial force when compared to typical rotary and impact drilling methods [36]. Controlled penetration experiments in simulated extraterrestrial regolith settings indicated that these drill bits can move freely in compacted soil layers without becoming stuck, which is a common problem with conventional drilling methods [37]. Furthermore, wear analysis of artificial excavation valves modeled after the locust ovipositor indicated that optimized material composition and structural adjustments could extend their service life, making these systems suitable for long-duration extraterrestrial exploration missions [36].

The application of locust biomechanics to mechanical drilling provides new opportunities for self-regulating and adaptable penetration devices. A study on the natural oviposition behavior of locusts demonstrated that these insects can dynamically modify the movement of the oviposition valve in response to varied substrate resistances [35]. This principle was included in the tests carried out [33], where the robotic drilling machine was outfitted with an instant feedback mechanism that can change the amplitude and frequency of the reciprocating action, allowing it to adapt to changing soil conditions. Comparative performance studies showed that the technique is effective at decreasing energy consumption while boosting penetration depth, making locust-inspired drills a feasible alternative for extraterrestrial exploration and subsurface sampling. While no extraterrestrial mission has yet adopted the locust-inspired drill, laboratory-scale robotic prototypes were tested on regolith simulants in 2022–2024, showing adaptive penetration and reduced wear. These studies remain in the TRL 4–5 range, i.e., component and subsystem validation in laboratory settings.

The creation of these biomimetic drilling devices marks a significant advancement in the design of efficient, lightweight, and adaptive penetration instruments. Studies have shown that the reciprocating drilling mechanism can beat existing approaches in energy-limited conditions by harnessing the locust ovipositor’s innate efficiency [36]. Continued breakthroughs in material science, robotics, and automation are projected to further enhance these systems and improve their suitability for extraterrestrial exploration and mining.

#### 3.1.3. Inchworm-Inspired Models

Research into inchworm-inspired drilling technology has resulted in important advances in extraterrestrial subsurface exploration. The inchworm’s drilling mechanism is based on a segmented body structure that expands and contracts in a regulated sequence, allowing for an efficient movement and penetration into granular and compacted soils [38,39]. As previously mentioned, unlike conventional rotary and percussion drills, which rely on external downward forces, the inchworm-inspired system uses internal actuation to generate forward motion while remaining stable in various substrates [40].

**Figure 9:**
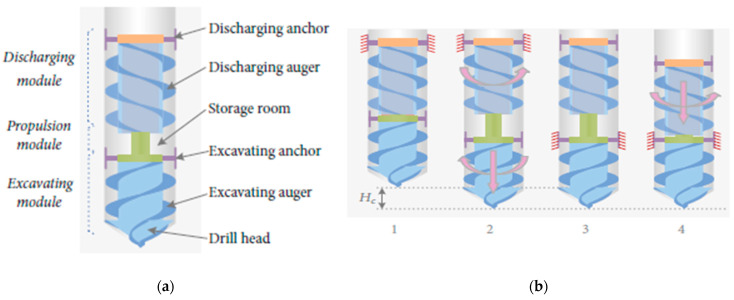
(**a**) Detailed structure of IBR. (**b**) IBR’s drilling principle [41].

Experimental studies have demonstrated that the inchworm boring robot (IBR) penetrated 510 mm at an average speed of 0.26 mm/s in the simulated regolith environment, as illustrated in Figure 9b [41]. This was performed without the use of an external discharge device, proving its capability to operate independently [38]. The research [40] suggested a propulsion module that coordinated alternate expansion and contraction between excavation and anchoring sections, resulting in a 25% increase in penetration efficiency over a static drill working under comparable conditions.

Further refinement of the drilling load model for lunar subterranean exploration revealed that the inchworm drilling robot uses less power due to its segmented drive approach. Tests performed in [41] for a lunar regolith simulant revealed that the adjusted drilling parameters dramatically reduced drag, allowing for deeper penetration while saving 30% more energy compared to traditional rotary drills. These studies demonstrated the effectiveness of inchworm-like architectures for energy-constrained extraterrestrial missions. The IBR design has been tested in terrestrial laboratory environments and microgravity parabolic flights [42] but has not yet been applied in space. Its modular design is currently undergoing further optimization to reduce the actuator bulk and improve the anchoring efficiency.

In addition to terrestrial testing, field trials in a microgravity setting were carried out to assess the adaptability of the IBR. Experiments have proven that the robot maintains traction and stability in low-gravity conditions using its cyclic anchoring system, limiting lateral displacement and optimizing depth advancement [39,42]. The use of real-time force sensing and adaptive control improved the inchworm drill’s performance by allowing it to dynamically modify the reciprocating amplitude based on the substrate density, reducing wasteful energy usage [38].

**Figure 10:**
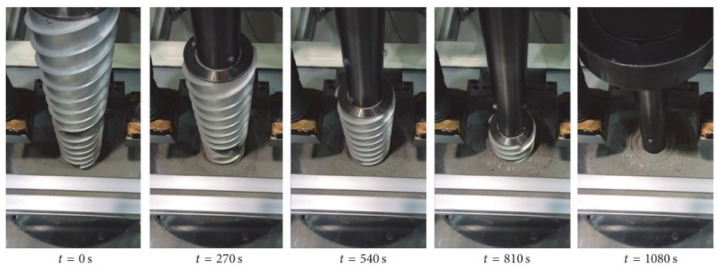
The process of drilling with IBR at certain time frames [41].

Recent improvements in inchworm-inspired extraterrestrial drilling included bidirectional drilling capabilities, allowing the device to drill down and then withdraw to retrieve a sample. Laboratory experiments have proven that the bidirectional module increases operational flexibility and makes underground exploration more efficient. The authors of [40] demonstrated that the inchworm drill may cut drilling time by 40% while retaining the integrity of granular and cohesive soils. The drilling process of GRC-3 lunar regolith simulant with a stainless steel IBR prototype is depicted in Figure 10 [41]. As research progresses, refinements in actuator design and soil interaction modeling are expected to further enhance the capabilities of these bioinspired systems, paving the way for deeper and more effective extraterrestrial subsurface investigations [41]. The IBR has undergone terrestrial and microgravity parabolic flight trials, warranting TRL-4, with further testing required for integration into a full robotic system.

#### 3.1.4. Clam-Inspired Models

**Figure 11:**
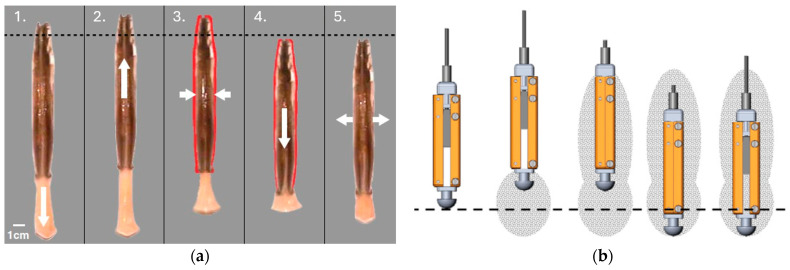
(**a**) Principal process of razor clam burrowing technology. (**b**) RoboClam working framework [44].

The burrowing mechanism of the razor clam (*Ensis directus*) has served as an essential model for the development of effective drilling systems. Razor clams (see Figure 11a) have evolved a dual-anchor mechanism that allows them to efficiently penetrate dense and hard surfaces like mud or compacted sand while consuming minimal energy [43]. This burrowing approach has been exploited to develop novel biomimetic drilling technologies that meet the constraints found in extraterrestrial environments, such as high energy costs, complicated regolith composition, and low gravity [44].

The clam’s burrowing model uses a coordinated cycle of valve contraction and expansion. Figure 11a depicts the initial stage in which the valve is opened to support the soil and establish a penetrating anchor. The foot then extends downward, entering the substrate, while the end anchor holds the clam in position. The subsequent constriction of the valve causes local fluidization, which occurs when the surrounding matrix transitions from a solid to a liquid state. This fluidized zone greatly reduces the drag, allowing the clams to migrate to deeper depths with less force [43,44,45,46].

In fluidized soils, burrowing energy is linearly linked to depth, while static soils exhibit a quadratic energy relationship. This efficiency is crucial for extraterrestrial applications with restricted energy supplies. The RoboClam, which applies these ideas to robotics, repeats the expansion–contraction cycle, resulting in efficient local fluidization and deeper penetration at lower energy costs [43].

Engineers, inspired by the clam’s methods, have created a number of biomimetic drills, such as the RoboClam shown in Figure 11b. This robotic system mimics the clam’s cyclic expansion and contraction movements to accomplish substrate fluidization on a small scale. The study [44] demonstrated that this robotic system has a great energy efficiency, with energy requirements growing linearly with depth, whereas for traditional drills the increase is quadratic. This feature makes the RoboClam ideal for lunar and Martian conditions, where energy conservation is crucial [47]. The RoboClam system has been applied successfully in Earth-based field trials [43,44], especially in underwater sediment tests and dry granular beds, but no full-scale implementation has been carried out in an extraterrestrial mission to date. In addition to subsurface sampling, the RoboClam’s ability to fluidize and penetrate compact regolith with a low energy input aligns well with ISRU needs, particularly the extraction of water ice or volatiles buried beneath the surface in permanently shadowed regions or polar deposits.

Recent developments in clam drilling technology have focused on improving the shape and control systems for usage in lunar regolith. Computational modeling with the discrete element method (DEM) revealed that certain design characteristics, such as longer anchors and optimized cone apex angles, might greatly improve the performance of the technology [45]. These designs reduced the energy required for penetration while retaining structural stability under various gravitational conditions. The rotational motion in the anchoring mechanism increased penetration efficiency by minimizing soil adhesion and improving torque transmission during drilling [48]. The RoboClam, though effective in underwater and loose sediment conditions, lacks vacuum and thermal validation, placing it at an estimated TRL of 3.

While the dual-anchor mechanism provides considerable benefits in terms of lower energy expenditures and increased penetration depth, converting this biological principle into a mechanical system faces difficulties. The difference in soil composition, as well as the need for strong and lightweight materials for extraterrestrial conditions, necessitates ongoing further developments and advancement of these technologies. Research into clam-inspired systems shows great potential for developing highly adaptive and efficient instruments for extraterrestrial exploration, site characterization, and resource extraction [44].

#### 3.1.5. Larva-Inspired Models

**Figure 12:**
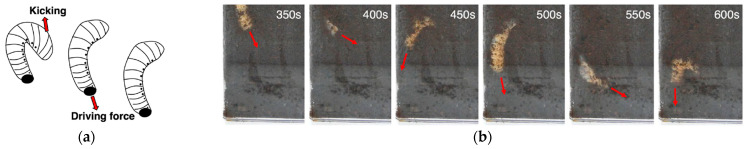
(**a**) Larva burrowing concept. (**b**) The dynamics of burrowing in soil [50].

The larval stage (see Figure 12a) of the scarab beetle (*Trypoxylus dichotomus*) offers an intriguing template for biomimetic drilling technology, particularly for use in areas with variable soil compositions and plentiful compacted debris. These larvae use a unique bimodal burrowing mechanism that adjusts their approach based on the stiffness of the surrounding substrate [49]. When exposed to a softer dirt, the larvae extend and retract their bodies in a linear pattern, comparable to peristaltic movement. In contrast, in tougher soils, they rotate and pivot, employing their body shape and rotational momentum to successfully remove soil [50].

Scarab beetle larvae have thick, segmented bodies that allow them to burrow alternately. Twisting and spinning their bodies produces local fluidization in compacted soil. The circular motion reduces mechanical drag by shifting the soil particles surrounding the larvae. This method reduces the force required to enter the dense matrix, allowing for a more efficient downward movement. The research [49] employed a two-dimensional surveillance system to show that larvae increased their rotation frequency in tougher soils, demonstrating their adaptability to changing levels of compaction. This rotating method not only displaces dirt but also provides stability during excavation, which is critical for robotic drilling technologies [50].

The scarab beetle larvae’s alternate burrowing approach prompted the development of biomimetic drills capable of operating in granular and compacted soils like those encountered on the moon and Mars [51]. Prototypes inspired by scarab larvae are at the early laboratory validation stages [50,51], and no terrestrial industrial deployment or space integration has occurred yet. By emulating the larvae’s capacity to alternate between linear and rotational movements, these drills can optimize energy consumption and adapt their manipulation tactics to the substrate’s mechanical properties. For example, a drill with this mechanism can transition between entering soft worn layers with low resistance and breaking through harder compacted layers utilizing rotational forces [50].

#### 3.1.6. Plant-Root-Inspired Models

**Figure 13:**
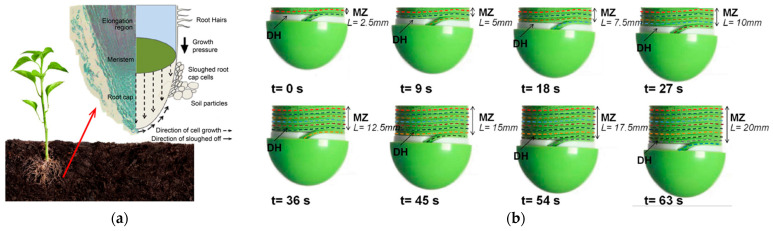
(**a**) Schematic representation of interaction between root cap, border cells, and soil particles [59]. (**b**) The growth process of the proposed robot [53].

Plant roots are a great model for the development of novel drilling and soil penetration technologies because of their energy-efficient techniques and adaptability to a variety of soil conditions. Because plant roots grow from the top using a special mechanism such as apical elongation via cell division and expansion, they effectively reduce friction and increase penetration efficiency in contrast to traditional penetration methods that rely on pushing or rotating forces. This led to the development of robotic systems that mimic nature’s strategies for soil testing and subsurface exploration in both terrestrial and extraterrestrial settings [52]. The elongation at the apex of the root where new cells are produced through mitosis is the main way that roots pierce the soil (Figure 13a). This effectively anchors the root and lowers frictional energy losses by allowing the root to grow while its mature sections stay motionless. By lubricating the soil–root interface, mucilage secretion and root cap cell sloughing further reduce resistance [52,53]. Researchers have been motivated by this and created robotic systems that mimic this growth mechanism. Studies have shown that the developed robot reduced the penetration forces by up to 70% compared to conventional drilling systems by simulating the cellular elongation and lubrication process [54].

Plant roots exhibit a remarkable morphological adaptability in response to the surrounding soil conditions. To improve anchorage and reduce penetration resistance, the roots may enlarge and produce additional lateral hairs when encountering compacted soils [55]. These adaptive characteristics have informed the development of robotic probes with flexible conical or tubular tips capable of modifying their geometry to navigate layered or granular substrates [56]. The study [57] demonstrated that bioinspired root-shaped probes can enhance penetration efficiency and reduce energy consumption, making them well-suited for extraterrestrial drilling and subsurface exploration.

The ability of plant roots to self-anchor in soil is a crucial characteristic that allows them to apply just enough force for sustained penetration. As seen in Figure 13b, this model has been implemented in robotic systems that employ deployable anchors or expandable structures to stabilize the apparatus while it is in use [54,58]. To replicate the function of lateral roots and hairs in providing stability, the robotic designs incorporated deployable layers or artificial root hairs, for example, and achieved deeper penetration while inhibiting backward motion [59]. Plant-root-inspired drilling systems have a wide range of applications, from geotechnical soil testing to subsurface exploration on asteroids, the moon, or planets like Mars [55]. Root-inspired probes have been tested in terrestrial soil analogs and soft robotics demonstrations [53], with some integration into agricultural sensing systems, but they are at the experimental stages for space deployment. These systems are also well-suited for ISRU scenarios that require a minimally invasive deployment of thermal sensors, volatile extractors, or embedded chemical probes, especially where anchoring and adaptive tip growth offer advantages over percussive methods [53,58]. Integration of the sensing capabilities into these bioinspired systems allows for real-time soil analysis, resource mapping, and structural stability assessments, expanding their utility in both scientific and industrial domains [60]. Plant-root-inspired systems, though conceptually promising for directional burrowing, currently remain at a TRL of 2–3, with only laboratory-scale soft robotic prototypes tested in simplified soil simulants. They have yet to be validated under environmental constraints relevant to space missions.

### 3.2. Horizontal Penetration

#### 3.2.1. Earthworm-Inspired Models

**Figure 14:**
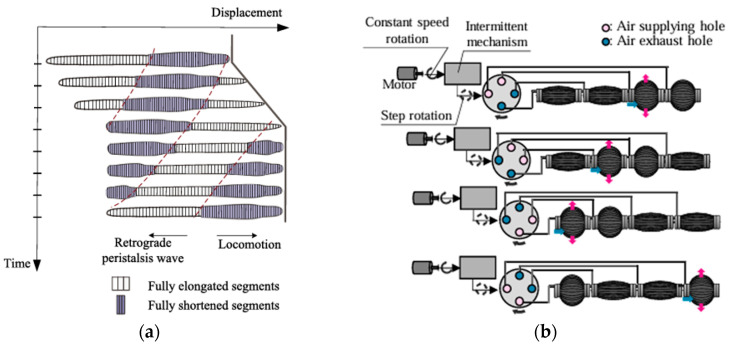
(**a**) Locomotion illustration of earthworms during burrowing [67]. (**b**) Appearance of robot locomotion and new pneumatic valve [63].

Peristalsis is a special kind of locomotion used by earthworms and is defined by coordinated contraction and relaxation of the circular and longitudinal muscles. This allows earthworms to propel some of their body segments forward while anchoring the other parts. The system of incompressible coelomic fluid that makes up their hydrostatic skeleton, which is surrounded by a muscular envelope, gives them structural support and allows for the shape changes required for movement and burrowing [61,62]. Based on this natural model, scientists have created robots that resemble earthworms and have modular pneumatic actuators that replicate peristaltic motion. As illustrated in Figure 14b, the research [63,64] presented a soft robotic system with axial and radial actuators that mimic the actions of the circular and longitudinal muscles of the earthworm allowing for movement through uneven underground pathways. In unstructured and diverse environments like extraterrestrial regolith, this method has proven to be particularly effective for maneuvering [42,65]. Earthworm-inspired soft robotic systems have undergone extensive terrestrial testing, including multi-terrain mobility experiments and underwater burrowing [63,66]. Some designs such as SEAVO-II have reached the field-validation stage in aquatic or semi-cohesive substrates, but no version has yet been space-qualified or tested under low-gravity conditions.

As illustrated in Figure 14a, another application of this approach is the SEAVO-II robot which has further modified the principles of earthworm locomotion for use in underwater drilling [66,67]. This system has achieved an enhanced torque transmission and reduced energy consumption by integrating seta-inspired gripping units and optimizing its auger design allowing for a deeper and more effective excavation [62,65]. Earthworm-inspired soft robots have demonstrated reliable motion in testbeds and some control autonomy, justifying a TRL of 4–5, though they remain untested in harsh space-like conditions.

#### 3.2.2. Mole-Inspired Models

**Figure 15:**
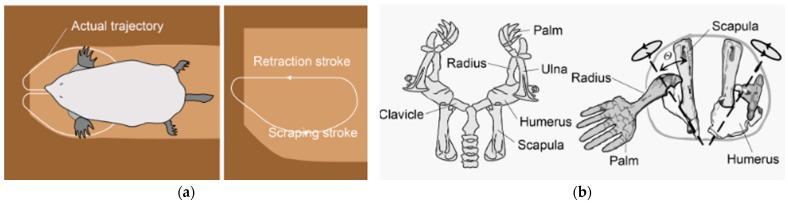
(**a**) Trajectory of mole’s forelimbs. (**b**) Detailed diagram of forelimb [72].

Engineers creating cutting-edge drilling systems have long been inspired by moles because of their exceptional burrowing skills. These creatures effectively dig tunnels larger than their bodies by using their strong forelimbs, as shown in Figure 15, and special anatomy as discussed below to break through compacted soil [68,69]. To overcome the difficulties of deep subsurface exploration in both terrestrial and extraterrestrial environments, bionic mole robots have been created by imitating these natural model mechanisms [70]. Moles use their powerful humerus clavicle and shoulder blades to produce the enormous force required for digging. To strengthen tunnel walls, their lateral forelimbs can be used to scoop up and compact soil [68,71]. This two-fold system guarantees the cave’s structural stability and effective soil removal. Moles can also remove compacted soil and make passages larger than their bodies thanks to their extremely flexible head and tooth movements [69,70].

**Figure 16:**
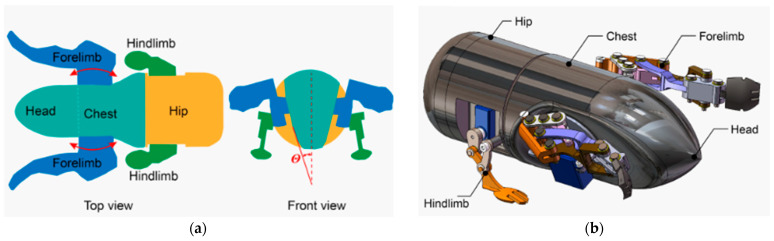
(**a**) Design concept of the mole-inspired robot. (**b**) 3D model of the robot [72].

The development of drilling robots with forelimb-like mechanisms and soil-removing tools was inspired by the burrowing behavior of moles. To replicate the motions of the mole’s shoulders and forelimbs, the Mole-Bot, for instance, has an expandable drill and debris removal system, as seen in Figure 16. The Mole-Bot has been validated in Earth-based testbeds simulating compacted soils and loose regolith, achieving a stable directional drilling with a reduced reaction force [69,70,73]. These systems are in the TRL 5–6 range, indicating a prototype validated in relevant environments but not yet qualified for flight with the potential for lunar deployment under development. No in situ extraterrestrial testing has yet occurred.

#### 3.2.3. Snake/Sandfish/Lizard-Inspired Models

**Figure 17:**
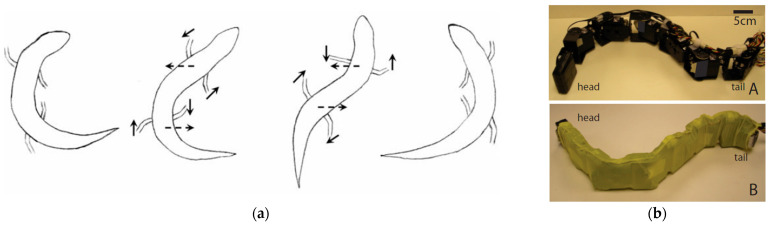
(**a**) Schematic movement of a sandfish [76]. (**b**) Sand-swimming robot wrapped in latex [74].

To effectively dig in granular environments, research into drilling methods modeled after snakes and lizards takes advantage of wave motion and friction anisotropy, providing a novel method for subterranean exploration. Motivated by the motions of snakes, lizards, and sandfish, the authors of [74,75] created a robotic system that can improve penetration efficiency and reduce drag in loose soils and regolith on the moon or Mars, as shown in Figure 17a. A substantial improvement over traditional rotary drilling methods, which usually experience excessive energy losses due to substrate drag, was demonstrated by experimental results showing that the sandfish-inspired robot had a wave efficiency of roughly 50%, meaning that half of its body motion contributed to forward motion [74]. Laboratory studies [76] have shown that the robot can swim undulatingly in granular media at up to 10 cm/s while still making steady progress in loose sand. This method is especially useful for extraterrestrial settings where traditional drilling methods have trouble with material clogging and compaction.

The ventral scales of certain snakes have directional friction coefficients that can reduce drag by up to 38% when moving forward according to research on the anisotropic friction characteristics of snake scales [77]. This model has been applied to a robotic drill that resembles a snake and uses differential friction to increase its motion efficiency, as illustrated in Figure 17b [75]. The model reproduced the ventral scale structure, and these robots could increase their propulsion efficiency in loose granular media while consuming 25% less power.

The creation of burrowing robots modeled after lizards has also produced encouraging outcomes. The ability of the eye-spotted skink to burrow into loose sand is restricted by its muscle strength but it can be optimized to lower the energy expenditure according to research on the animal’s controlled burial abilities [78]. In comparison to conventional mechanical digging techniques, this strategy reduces the energy requirement for digging by 30% when implemented in a robotic system. Adding an active body undulation to current robotic platforms can increase their locomotion efficiency by 40% while requiring less power according to recent developments in sandfish-inspired robotic burrowing techniques [79]. Sandfish-inspired and snake-like robots have been successfully tested in laboratory-scale granular beds and in regolith simulant tests [75,80], with reported gains in drag reduction and directional control. However, they have not yet progressed to extraterrestrial mission integration, and the fabrication of scale-resilient anisotropic surfaces remains a key technological barrier. Since an efficient subsurface sampling is essential for scientific research, this has significant ramifications for extraterrestrial exploration.

Additionally, by incorporating the resistance theory into the robot control system, the prediction model was enhanced and real-time adaptation to various substrate conditions was made possible [79]. The exploration and excavation methods for both terrestrial and extraterrestrial applications are evolving due to the use of biomechanics in drilling technology inspired by snakes and lizards. The ability to move through loose soils efficiently while minimizing drag and power consumption makes these biomimetic systems appealing for extraterrestrial missions [80]. The research [76] advanced these technologies by incorporating adaptive motion techniques and sensor-based feedback systems to increase their performance in harsh conditions.

## 4. Discussion

To assist in comparing the various approaches discussed in the section above, Table 1 below summarizes the key biological inspirations, core drilling/burrowing principles, reported advantages, primary challenges, and example references. In Table 1, the “Most Suitable Environment” is defined based on a qualitative evaluation of each system’s mechanical requirements, anchoring strategies, actuation methods, and reported testing conditions.

Each mechanism presented in Table 1 offers distinct advantages in energy reduction, debris management, or anchoring. Still, these bioinspired strategies often face scale-up challenges, especially for space missions that demand ruggedized materials and robust autonomous control. Section 5 discusses these overarching challenges and connects them to technological readiness for future deployment. While most bioinspired drilling concepts are currently in the experimental or prototype stages, several systems, particularly those based on wood wasps and clams, have shown significant promise in Earth-based trials using regolith simulants. No bioinspired drill has yet been deployed in an active space mission, but increasing interest from agencies such as NASA and ESA, combined with advancing TRL levels, suggests a strong potential for near-future adoption. Altogether, while horizontal bioinspired systems have achieved promising terrestrial validation, they generally remain at the prototype stage, with future advancements needed in actuator miniaturization, control systems, and material robustness for space-grade deployment.

Some trade-offs become visible across the considered biomimetic systems. Bioinspired solutions, such as those based on earthworms or sandfish with their low anchoring force and distributed actuation characteristics, are particularly suitable for microgravity situations and represent an optimal solution for asteroids or small body exploration. In contrast, mole-, wood wasp-, and scarab beetle-based designs require a higher interaction force and more complex mechanics systems to be driven or controlled but can achieve deeper penetration and therefore may be better suited for operations on Mars or the moon. The TRL levels for various models differ widely; mole- or wasp-based designs, for instance, have already been tested in regolith simulants while plant-root or razor clam types are mainly conceptual. Peristaltic or undulatory systems are particularly attractive, as they provide an intrinsic mechanical autonomy and fault tolerance at the expense of a slower excavation rate. In the end, selection of the drill also involves a trade-off between environmental compatibility, mission lifetime, and energy supply limitations, as well as TRL, and this may suggest a hybrid approach or modular strategy for upcoming planetary drilling systems.

Although many of these bioinspired drilling systems demonstrate interesting design advantages, the vast majority of the prototypes have not been tested outside the laboratory. A significant challenge for many of these systems is the dependence on Earth-based conditions that do not adequately reproduce the vacuum, extreme swings in temperature, and reduced gravity of space. For example, razor clam- or root-inspired designs have thus far only been tested in wet or granular terrestrial substrates that are quite distinct from the compacted lunar regolith or dry Martian regolith. Wear and fatigue have also been insufficiently studied for soft-bodied designs such as earthworm-inspired actuators. Mechanical anchoring and actuation also are still unresolved for the free-flying scenario, particularly so on small bodies like asteroids. In addition, most works do not have an explicit quantification of power penetration ratios and failure rates or evaluation of the long-term operation performance. These deficiencies point to the necessity of greater mechanical validation, along with long-term performance testing and environmental simulation, prior to bioinspired systems being space-qualifiable.

Also, it is worth noting that there is a difference between the biological function of drilling (e.g., in terms of movement, protection, reproduction) and the engineering purpose of space drilling which is largely intended for sample gathering or resource extraction. Many animals burrow for habitat construction or locomotion, and not all of these modes can be readily adapted to the robotic drilling and sample collection problem. Of the methods reviewed in this paper, results such as those from the inchworm-inspired boring robot and wood wasp-inspired reciprocating drills show the most promise in terms of extracting cores or taking subsurface samples. The others, such as sandfish- or mole-inspired systems, may be more suitable for transporting, anchoring, or regolith dislodging but would necessitate separate devices for sample containment. Therefore, a combination strategy which includes both a propellant production mechanism and sampling ability may be required for efficient ISRU missions.

## 5. Challenges and Limitations

Although biomimetic drilling technologies have made encouraging strides, a number of obstacles and restrictions still need to be overcome before they can be used in extraterrestrial applications. The primary concern is biomimetic mechanisms’ scalability. Although biological systems function well at their natural scale, it is extremely challenging for engineers to replicate these mechanisms and their performance at the macroscale for extraterrestrial drilling [27,81]. The intricate morphologies of clam foot dynamics, insect ovipositors, and serpentine frictional adaptations need to be translated into strong long-lasting materials that can survive the challenging conditions of extraterrestrial environments, such as asteroids, the moon, and Mars.

One of the biggest technological challenges is producing parts that are both strong and lightweight [13,82]. Power limitations and energy efficiency present another significant obstacle. Bioinspired drills use less power than conventional drilling techniques but there are still severe energy restrictions in extraterrestrial environments that must be met. As stated in the paper [83], to ensure continuous operation the mission planners must optimize power sources like solar panels or radioisotope thermoelectric generators. Additionally, it is still difficult to create effective actuators that can replicate biological motions without using a lot of power [70].

Gravitational force is also an important parameter affecting the development of drilling system designs and performance. Given the baseline gravity of 9.81 m/s on Earth, where aircrafts and spacecrafts are designed to function, the environments on both Mars (3.7 m/s) and the moon (1.6 m/s) can pose challenges for terrestrial equipment and the humans who use it [84]. This wide range of variation has a large influence on the penetration mechanics, requirements for anchoring, and behavior of cuttings during drilling. The systems designed for low-gravity locales must make up for the absence of the normal force, typically via addition of anchoring arms, counter-rotating devices, or percussive action to keep things from just skipping along the surface.

The wear resistance and material adaptability are extremely limited. The research [29] underlined that robotic drills must function with little maintenance over extended missions in contrast to living organisms that constantly regenerate their structures. Extraterrestrial regolith like Martian soil or lunar dust is abrasive, which speeds up tool wear and may result in a decreased performance. Research on coatings or self-healing materials modeled after biological systems may help to mitigate this issue [27].

Autonomous control and flexibility are also important, i.e., when the organisms receive feedback from their surroundings, they automatically modify their penetration tactics. Advanced sensor integration, real-time computational modeling, and artificial intelligence-driven decision-making are necessary for the robotic systems to achieve comparable adaptability [82,85]. Current biomimetic drills still have trouble reacting dynamically to changes in the substrate and further research is required to improve their autonomous navigation and terrain analysis.

The implementation of biomimetic drilling systems is made more difficult by environmental limitations like drastic temperature swings, low gravity, and vacuum conditions. One study [85] showed how the performance of mechanical parts may be hampered by the presence of electrostatically charged dust particles, thermal fluctuations, and the lack of atmospheric pressure. Extensive testing and material innovation are necessary to ensure the long-term reliability of biomimetic drilling systems under such circumstances.

The scaling of bioinspired drilling systems from laboratory prototypes to space-flight-ready tools presents a major engineering challenge. Many current designs demonstrate efficiency at millimeter or centimeter scales but suffer a performance loss, mechanical instability, or actuator overload when scaled up [81,86]. Biological systems often operate under soft-bodied, distributed actuation schemes that are hard to replicate in larger, rigid robotic counterparts. Furthermore, the energy efficiency and control strategies of the natural models must be fundamentally re-engineered when increasing the size of the engineered models, especially for operation under planetary gravity and regolith interaction forces. As stated in the paper [87], the overall size of the robot should not exceed 2.5 m. As such, validating bioinspired mechanisms at mission-relevant scales remains a critical and largely unresolved challenge.

One aspect that has been largely ignored in the development of biomimetic drilling systems is that of radiation hardness. Unlike living systems which inhabit the Earth’s magnetosphere, robotic drills must endure long-term exposure to cosmic radiation and solar particle events. This may cause wastage of actuators, sensors, and embedded electronics. Soft or flexible materials such as polymers, elastomers, etc., used in soft robotics (e.g., inchworm- or root-inspired systems) can either turn stiff/brittle or electrostatically charged under radiation, thus completely losing their motion control. It is an important task to study the shielding demands and radiation-hardened control architectures for these systems in the future.

Furthermore, environmental drilling limitations should be considered for bioinspired mechanisms. The penetration depth of conventional space drills is constrained by the depth to which the compacted regolith or ice can be penetrated with a reasonable amount of energy and mechanical complexity, reaching an effective depth of 2–3 m. Nature’s analogs have differing penetration capabilities; for example, clam-like fluidization can greatly reduce drag but might be limited by particle cohesion when the regolith is frozen, and inchworm- and wasp-inspired systems have shown promise over consolidated media with imposed axial loads. However, difficulties arise in the broad application of these approaches to diverse extraterrestrial situations with high-density rocky media, severe dehydration conditions, or volatile-rich horizons. This approach requires that such drilling strategies be selectively used depending on whether the substrate is hard or soft, the required depth of drilling is deep or shallow, and an energy budget exists for the entire mission (see Table 2).

Tool wear is a critical issue for extraterrestrial scenarios with little redundancy and material substitution possible. These biological systems generally use tissue that can regenerate (working lifetime) the insect ovipositor or work around a limited lifetime, whereas the space system needs to have high durability against cyclic operation. Reciprocating mechanisms, such as those based on the wood wasp or locust, are vulnerable to cyclic stress concentrating at teeth and joints with occasional abrasive wear when interacting with simulants such as JSC-Mars-1 or lunar dust analogs, but worm-like penetration minimizes force bias which may be expected to lower the rate of wear. Clam-based fluidization minimizes physical interactions with particles, theoretically providing long-term superiority. However, there have been limited comparisons of the wear rates across these three models, and additional material testing on simulant regolith is required. The development of wear-resistant composites, diamond-coated drill tips, or self-healing polymers would greatly alleviate this problem. A comparative wear susceptibility summary is given in Table 3.

## 6. Future Directions and Opportunities

The continuous application of bioinspired concepts to cutting-edge engineering solutions is key to the future of nature-inspired drilling technologies. As recommended [88,89], future research requires more of a focus on enhancing biomimetic actuators and structures to boost their robustness, effectiveness, and adaptability in extraterrestrial environments [13]. To increase penetration efficiency and operational flexibility, hybrid drilling systems that combine impact or rotational mechanisms with biomimetic reciprocating motion are a promising approach [85]. As space agencies increasingly prioritize the ISRU for sustainability and mission autonomy, these hybrid bioinspired models can offer a pathway to the successful extraction of water, regolith, or other materials without the excessive power, vibration, or system mass typical of traditional drills [80].

It is anticipated that further advancements in material science will be crucial in resolving concerns associated with the lifespan of the engineered models and their wear resistance. The research [29] recommends using nanoengineered coatings, advanced composites, and self-healing polymers to greatly increase the drilling tool’s service life. These materials draw inspiration from biological regeneration mechanisms and may allow for in situ maintenance negating the need for extensive replacement or repair as stated by [70].

Adaptive control systems powered by artificial intelligence present yet another significant opportunity. Biomimetic drills will be able to independently modify their drilling strategy based on substrate composition and resistance by implementing machine learning algorithms that evaluate real-time sensor data [85]. These advancements have the potential to increase operational autonomy, decrease the need for direct human intervention, and boost the effectiveness of distant extraterrestrial missions. Another fascinating avenue for bioinspired drilling is the incorporation of swarm robotics technologies. The study [90] showed that several smaller biomimetic drills can cooperate to replicate the collective behavior of burrowing insects or digging animals rather than using a single large drilling device. This strategy could increase extraterrestrial exploration missions’ resilience and adaptability to different terrains by increasing their efficiency, redundancy, and adaptability.

Biomimetic drilling technology may eventually be used for purposes other than extraterrestrial exploration alone. Where traditional drilling techniques encounter comparability difficulties, the ideas discussed for extraterrestrial application can also be used for resource extraction, deep-sea exploration, and underground construction on Earth [65,89]. The research [51,80] stated that biomimicry robotics and material science will continue to spur innovation and create new avenues for intelligent, sustainable, and effective subterranean exploration. These insights align with the broader trends in biologically inspired systems in space applications, as reviewed in [91], highlighting their potential for adaptability, energy efficiency, and terrain compliance.

## 7. Conclusions

This paper has reviewed a variety of biomimetic drilling systems which have been inspired by biological mechanisms like peristalsis, undulation, abrasion, burrowing, and penetration. Inspired by organisms (earthworms, moles, and wood wasps) and presenting nature-inspired robot designs (sandfish, razor clams, and plant roots) developed for planetary subsurface access, these mechanisms are promising in terms of providing a sustainable solution to long-standing challenges involving planetary subsurface access, such as energy efficiency, debris management, and anchoring.

The comparative analysis shows that earthworm/sandfish-inspired undulatory distributed actuation systems appear particularly appropriate for use in low-gravity scenarios because they require little anchoring and are also flexible within a granular material. On the other hand, more aggressive or mechanically sophisticated devices that resemble mole-like claws or wasp-like drills feature a higher penetration performance and would be more suitable for dense regolith such as that present on the moon and Mars. However, most of these models are at an early TRL development stage and not yet validated outside laboratory conditions.

All considered systems have common shortcomings, namely, in situ tests under space-like thermal and gravitational conditions have not been conducted yet, the issue of long-term durability has not been resolved, and specific data on the wear, energy-per-penetration efficiency of the device, and autonomy of the system are scarce. These gaps require future research that should be based on a comprehensive environmental simulation, long-term testing, and integration of adaptive control systems.

Integrating the mechanical penetration efficiency of wood wasp-inspired drills with the distributed anchoring and peristaltic motion capabilities of inchworm-based locomotion could provide a mission-flexible architecture. Additionally, such a system could be applied as a structural support for deep regolith penetration and retain mobility and anchorage stability in low-gravity environments. While space agencies are progressing towards more ambitious ISRU as well as deep drilling missions, biomimetic systems represent an interdisciplinary approach that offers unexplored potential. Exploiting this opportunity, however, will require us to span the distance from biological inspiration to engineered implementation by stringent testing and gradually building up the system.

## Figures and Tables

**Table 1:** Comparison of bioinspired drilling mechanisms.

Organism	Drilling Principle	Key Advantage	Main Challenge	Most Suitable Environment
Wood Wasp	Dual-valve reciprocation	50% reduction in overhead force [12]	Material durability for reciprocating parts [25]	Moon, Mars
Locust Ovipositor	Asymmetric reciprocation	Lower frictional drag, adaptability [36]	Balancing valve wear in compacted soils [37]	Moon, Mars
Inchworm	Segmented expansion/contraction	Reduced power consumption, stable traction [38]	Complex actuation and control [41]	Moon, Mars
Razor Clam	Localized fluidization, dual anchor	Up to 90% drag reduction, linear energy scaling [44]	Reliable valve sealing, mechanical complexity [45]	Moon, Asteroids
Scarab Larva	Dual-mode (linear vs. rotational) burrowing	Broad substrate adaptability [49]	Smoothly switching between modes [50]	Mars
Plant Root	Tip-based growth, mucilage lubrication	Up to 70% penetration force reduction [54]	Slow penetration rates, complex biomimicry [56]	Asteroids
Earthworm	Peristalsis with hydrostatic skeleton	Flexible in heterogeneous soils [37]	Sealing actuators to prevent dust intrusion [65]	Asteroids
Mole	Forelimb scooping and tunnel reinforcement	Efficient soil displacement [70]	Designing robust scooping actuators [73]	Mars
Sandfish/Snake/Lizard	Undulatory locomotion, frictional anisotropy	40%+ improvement in granular penetration [74]	Fabricating anisotropic friction surfaces [79]	Moon, Asteroids

**Table 2:** Comparison of bioinspired drills.

Biological Inspiration	Max Penetration Depth	Test Condition	Power Consumption	Sampling Capability	Autonomy Potential	Primary Limitation
Wood Wasp (DROD) [12,13]	2.0 m	Granular regolith	3–5 W	Yes	Open loop control	Bit wear, actuator complexity
Locust Ovipositor [33,37]	1.2–1.5 m	Dense cohesive soils	~6–8 W	Partial	Feedback driven valve modulation	Mechanical durability under friction
Inchworm (IBR) [40,41]	510 mm	Granular and compacted soil	9.8 W	Yes	Adaptive anchoring and motion	Large size, actuator weight
Razor Clam (RoboClam) [44]	60 cm	Fluidizable soils only	1.5–2.5 W	No	Simple cyclic actuation	Requires saturated/cohesive matrix
Scarab Larva [50,51]	30–50 cm	Compact/loose dual-layered soils	~5–10 W	Limited	Mode-switching control	Unclear switching efficiency
Plant Root [53,54]	20–40 cm	Soft granular only	1–2 W	Yes	Growth-driven control	Penetration speed (~1 mm/min)
Earthworm [65]	30 cm	Heterogeneous soils	5.6–7 W	No	Limited locomotion sequences	No soil extraction function
Mole [73]	~1 m	Hard/abrasive soils	12–20 W	Limited	Lack of real-time path planning	Complex forelimb actuator
Sandfish/ Snake/Lizards [74,76]	~0.5 m	Loose sand; dry regolith	4–6 W	No	Wave-based locomotion	No sampling ability, traction loss in dense soil

**Table 3:** Comparative wear risk and durability of bioinspired drills.

Mechanisms	Tool-Soil Contact Mode	Estimated Wear Rate	Validated Lifetime	Material Tested	Wear Mitigation	Biological Analogy
Wood Wasp (DROD) [25,29]	High (metal teeth into simulant)	~0.1 mm per 50 cm drilled	~20 full insertions	Steel, carbide	Replaceable bits, diamond coating in tests	Ovipositor regenerates in cycles
Locust Ovipositor [36]	Moderate (asymmetric valve wear)	~15–20% mass loss in 100 cycles	~100 cycles	Hardened steel, polymeric interfaces	Asymmetric modulation reduces wear	Short-term biological use
Inchworm (IBR) [40,41]	Low–moderate (distributed anchors)	<0.05 mm per 50 cm	50+ cycles in lab tests	Aluminum, PTFE interfaces	Minimal force per contact area	Large size, actuator weight
Razor Clam (RoboClam) [44]	Very low (fluidization reduces drag)	Unmeasured in dry regolith, negligible in wet	Unknown in dry media	ABS, rubber composites	Fluidized zone minimizes contact	Shell cycles reduce fatigue
Scarab Larva [50,51]	Moderate (torsional and linear)	No quantified rate reported	Unknown	Rubber and rotational appendages	Twisting mode reduces local pressure	Adaptive motion offsets impact
Plant Root [53,54]	Low (tip-based pushing, not cutting)	Unmeasured, negligible	Unknown	Silicone and flexible polymers	Mucilage analogs reduce friction	Grows by cell division at tip
Earthworm [63]	Low–moderate (muscle actuator wear)	~0.05 mm actuator loss per 25 cm	~25 cycles per actuator set	Silicone bladder, pneumatics	Periodic actuator sealing	Coelomic fluid and muscle reconditioning
Mole [70]	High (forelimb mechanical scraping)	Fast abrasion in compact soil	~5–10 drills before maintenance	Hardened steel, aluminum alloys	Hardened tips, lubrication	Bone–muscle actuation resists fatigue
Sandfish/ Snake/Lizard [77]	Low–moderate (sliding friction)	~38% reduction with anisotropic surfaces	Not reported	Latex skin, embedded ridges	Ventral scale emulation	Skin shedding renews friction layer

## Data Availability

No new data were created or analyzed in this study.
